# The neurovascular relation in oxygen-induced retinopathy

**Published:** 2008-12-26

**Authors:** James D. Akula, Julie A. Mocko, Ilan Y. Benador, Ronald M. Hansen, Tara L. Favazza, Tanya C. Vyhovsky, Anne B. Fulton

**Affiliations:** 1 Department of Ophthalmology, Children’s Hospital Boston, Boston, MA; 2 Department of Ophthalmology, Harvard Medical School, Boston, MA

## Abstract

**Purpose:**

Longitudinal studies in rat models of retinopathy of prematurity (ROP) have demonstrated that abnormalities of retinal vasculature and function change hand-in-hand. In the developing retina, vascular and neural structures are under cooperative molecular control. In this study of rats with oxygen-induced retinopathy (OIR) models of ROP, mRNA expression of vascular endothelial growth factor (*VEGF*), semaphorin (*Sema*), and their neuropilin receptor (*NRP*) were examined during the course of retinopathy to evaluate their roles in the observed neurovascular congruency.

**Methods:**

Oxygen exposures designed to induce retinopathy were delivered to Sprague-Dawley rat pups (n=36) from postnatal day (P) 0 to P14 or from P7 to P14. Room-air-reared controls (n=18) were also studied. Sensitivities of the rod photoreceptors (*S*_rod_) and the postreceptor cells (*Sm*) were derived from electroretinographic (ERG) records. Arteriolar tortuosity, *T*_A_, was derived from digital fundus images using Retinal Image multi-Scale Analysis (RISA) image analysis software. mRNA expression of *VEGF_164_*, semaphorin IIIA (*Sema3A*), and neuropilin-1 (*NRP-1*) was evaluated by RT–PCR of retinal extracts. Tests were performed at P15–P16, P18–P19, and P25–P26. Relations among ERG, RISA, and PCR parameters were evaluated using linear regression on log transformed data.

**Results:**

*Sm* was low and *T*_A_ was high at young ages, then both resolved by P25–P26. *VEGF_164_* and *Sema3A* mRNA expression were also elevated early and decreased with age. Low *Sm* was significantly associated with high *VEGF_164_* and *Sema3A* expression. Low *S*_rod_ was also significantly associated with high *VEGF_164_*. *S*_rod_ and *Sm* were both correlated with *T*_A_. *NRP-1* expression was little affected by OIR.

**Conclusions:**

The postreceptor retina appears to mediate the vascular abnormalities that characterize OIR. Because of the relationships revealed by these data, early treatment that targets the neural retina may mitigate the effects of ROP.

## Introduction

High oxygen has long been associated with pathologic retinal vascular abnormalities [1–4], the clinical hallmark of retinopathy of prematurity (ROP) [5]. But persistent dysfunction of the neural retina is increasingly recognized as an essential component of the ROP disease process. Persistent deficits in rod and rod-bipolar cell sensitivity are detectable years after acute ROP has resolved [6–11]. The severity of these neural deficits varies with the degree of the antecedent vascular disease. The abnormalities in retinal blood vessels that characterize ROP appear within a narrow preterm age range when the developing rod outer segments are elongating rapidly, accompanied by an increase in the rhodopsin content of the retina and burgeoning energy demands in the photoreceptors [12].

In rat models of ROP, rod photoreceptor dysfunction antedates [13] and predicts [14] the subsequent retinal vascular abnormalities, and persists after their resolution [14,15]. The mechanisms that underpin these phenomena remain to be elucidated.

The postreceptor retina, too, is affected by ROP. Moreover, postreceptor sensitivity recovers hand-in-hand with resolution of the retinal vascular abnormalities [14,15]. Indeed, the retinal vasculature and the postreceptor neural retina are in close physical proximity, are immature at the same ages, and develop together by processes termed angiogenesis and neurogenesis, respectively. It stands to reason that there must be “remodeling” [16] mechanisms that mediate the neurovascular congruency. Molecules, called growth factors, that cooperatively control both angiogenesis and neurogenesis [17] are abundant in the developing retina, and, thus, are candidate mediators of the neurovascular interplay documented in ROP. We studied mRNA expression of neurovascular growth factors in rat models of ROP.

From the angiogenesis pathway, we selected vascular endothelial growth factor (*VEGF*). *VEGF* is essential for normal blood vessel growth in the developing retina [18,19] and is implicated in the pathogenesis of vasoproliferative retinal diseases like ROP [20–22]. From the neurogenesis pathway, we selected semaphorin because it acts as an axon growth cone guidance molecule [23] involved in postreceptor retinal development and likely in plasticity and stabilization (as during recovery from an insult) of postreceptor signaling [24]. We also assayed neuropilin-1 (*NRP-1*), a coreceptor for both *VEGF* [25,26] and semaphorin [27,28]. *NRP-1* is expressed both in vascular endothelial cells and in retinal neurons [29], including in the progenitors of photoreceptors [30]. That *NRP-1* mediates both neural and vascular development by the competitive binding of two disparate ligand families, *VEGF* and semaphorin, supports the hypothesis that retinal neurogenesis and angiogenesis are inseparably linked [31,32]. This is further supported by the observation that *VEGF*, semaphorin, and *NRP-1* are expressed in temporally and spatially overlapping domains during retinal development [24,33]. In addition, semaphorins play a direct role in angiogenesis not mediated by neuropilin [34–36]. Thus, as has been documented in oncogenesis where semaphorins have a demonstrated role in the development of vascular supply [37], semaphorins likely play a role in the development of retinal vasculature as well as retinal neurons.

We selected specific isoforms of *VEGF* and semaphorin based upon the degree of specificity in *VEGF*/neuropilin and semaphorin/neuropilin binding affinity and activity. *NRP-1* is specifically sensitive to the *VEGF_164_* isoform (*VEGF_164_*) [26], the ortholog of primate *VEGF_165_*, and of the semaphorin family of ligands, has highest affinity for semaphorin IIIA (*Sema3A*) [38]. Herein, we studied the mRNA expression of these growth factors in rats with oxygen-induced retinopathies (OIR) that model the gamut of severity of human ROP [14]. In every rat, we also obtained numeric measurements of rod photoreceptor and postreceptor neural function and of blood vessel abnormality.

## Methods

### Subjects

This study followed a cross-sectional design and used 54 Sprague-Dawley albino rats (Charles River Laboratories Inc., Worcester, MA) from nine litters. Rats were assigned to one of three groups (n=18), either of two OIR paradigms or controls. Tests of neural function, vascular abnormality, and growth factor mRNA expression were performed at postnatal day (P) 15–P16, P18–P19, or P25–P26. P15–P16 is immediately after the induction of ROP. At P18–P19, the vascular abnormalities are quite marked [14,15]. At P25–P26, vascular abnormalities are rapidly resolving [14,15]. All experiments were conducted according to the ARVO Statement for the Use of Animals in Ophthalmic and Vision Research with the approval of the Animal Care and Use Committee at Children’s Hospital Boston.

### Induction of retinopathy

As described previously in detail [14], OIR was induced by placing pups and dam in an OxyCycler (Biospherix Ltd., Lacona, NY) and exposing them to one of two different oxygen regimens designed to produce a range of effects on the retinal vasculature and the neural retina. The first OIR regimen, the 50/10 model, involved exposure to alternating 24 h periods of 50±1% and 10±1% oxygen from P0 (the day of birth) to P14 [39]. The second OIR regimen, the 75 model, was exposure to 75±1% oxygen from P7 to P14. Controls were reared in room air (21% oxygen). While the 50/10 model reliably produces peripheral neovascularization [39] and tortuosity of the posterior retinal arterioles [14,15], rat models created by exposure to continuous high oxygen, similar to our 75% model, reliably produce tortuosity of the central retinal vasculature [14], but infrequently produce peripheral neovascularization [40,41]. Both OIR paradigms target the ages at which the rod outer segments are forming and when the rhodopsin content of the retina is rapidly increasing. Of note, at birth, about 50% of the eventual rod photoreceptors are differentiated; however, only a small number of the second-order bipolar cells are present. By P9, differentiation of both rod photoreceptor and postreceptor neurons is essentially complete [42]. But rod outer segment development and vascular coverage remain incomplete.

### Analyses of neural function

#### Calibration of stimuli

Function of rod photoreceptor and postreceptor retinal neurons was assessed by electroretinography (ERG). The stimuli were delivered using an Espion *e^2^* with ColorDome Ganzfeld stimulator (Diagnosys LLC, Lowell, MA). The rate of photoisomerization per rod (R*) for the green LED flash was calculated by measuring the flux density incident upon an integrating radiometer (IL1700; International Light, Newburyport, MA) positioned at the location of the rats’ cornea, and following the procedures detailed by Lyubarsky and Pugh [43]. The LED was treated as monochromatic with λ equal to 530 nm. The intensity of the flash was given by

$$i\left(\lambda \right)=Q\left(\lambda \right)\times T\left(\lambda \right)\times \frac{a_{\text{pupil}}}{a_{\text{retina}}}\times a_{rod}\left(\lambda \right)$$*Equation* 1

where *i*(λ) is the number of photoisomerizations per rod (R*) elicited by the flash, *Q*(λ) is the calculated photon density at the cornea, *T*(λ) is the transmissivity of the ocular-media and pre-receptor retina (approximately 80% at 530 nm [44]), and *a*_pupil_, *a*_retina_, and *a*_rod_(λ) are respective estimates of the area of the dilated pupil (approximately 20 mm^2^ [45]), the area of the retinal surface (approximately 50 mm^2^ [46]), and the end-on light collecting area of the rod photoreceptor (approximately 1.5 µm^2^ at 530 nm). *a*_rod_(λ) takes into account the length of the outer segment, the absorption spectrum of the rod, and the optical density of the photopigment, as well as the radius of the photoreceptor [47]. Since several of these parameter values are unknown for the rat rod that is affected by OIR, stimuli are expressed as the expected values in adult control rats. We calculated *Q*(λ) by

$$Q\left(\lambda \right)=\lambda \times \frac{P_{\text{λ}}}{h\times c}$$*Equation* 2

where *P*_λ_ is the radiant flux (watts), *h* is Planck’s constant, and *c* is the speed of light [48]. To evaluate the intensity of “white” xenon arc flashes, we recorded an intensity series with interspersed green and white flashes and estimated the equivalent light based on the shift of the stimulus/response curves for the scotopic b-wave.

#### Preparation

Prior to ERG testing, rats were dark adapted for a minimum of 2.5 h. Preparations were made under dim red illumination. Subjects were anesthetized with a loading dose of approximately 75 mg∙kg^−1^ ketamine and approximately 7.5 mg∙kg^−1^ xylazine, injected intraperitoneally. This was followed, if needed, by a booster dose (50% of loading dose) administered intramuscularly. The pupils were dilated with a combination of 1% phenylephrine hydrochloride and 0.2% cyclopentolate hydrochloride (Cyclomydril; Alcon, Fort Worth, TX). The corneas were anesthetized with one drop of 0.5% proparacaine hydrochloride (Alcon). A Burian-Allen bipolar electrode (Hansen Laboratories, Coralville, IA) was placed on the cornea and the ground electrode was placed on the tail.

#### Analysis of rod function

**Figure 1:**
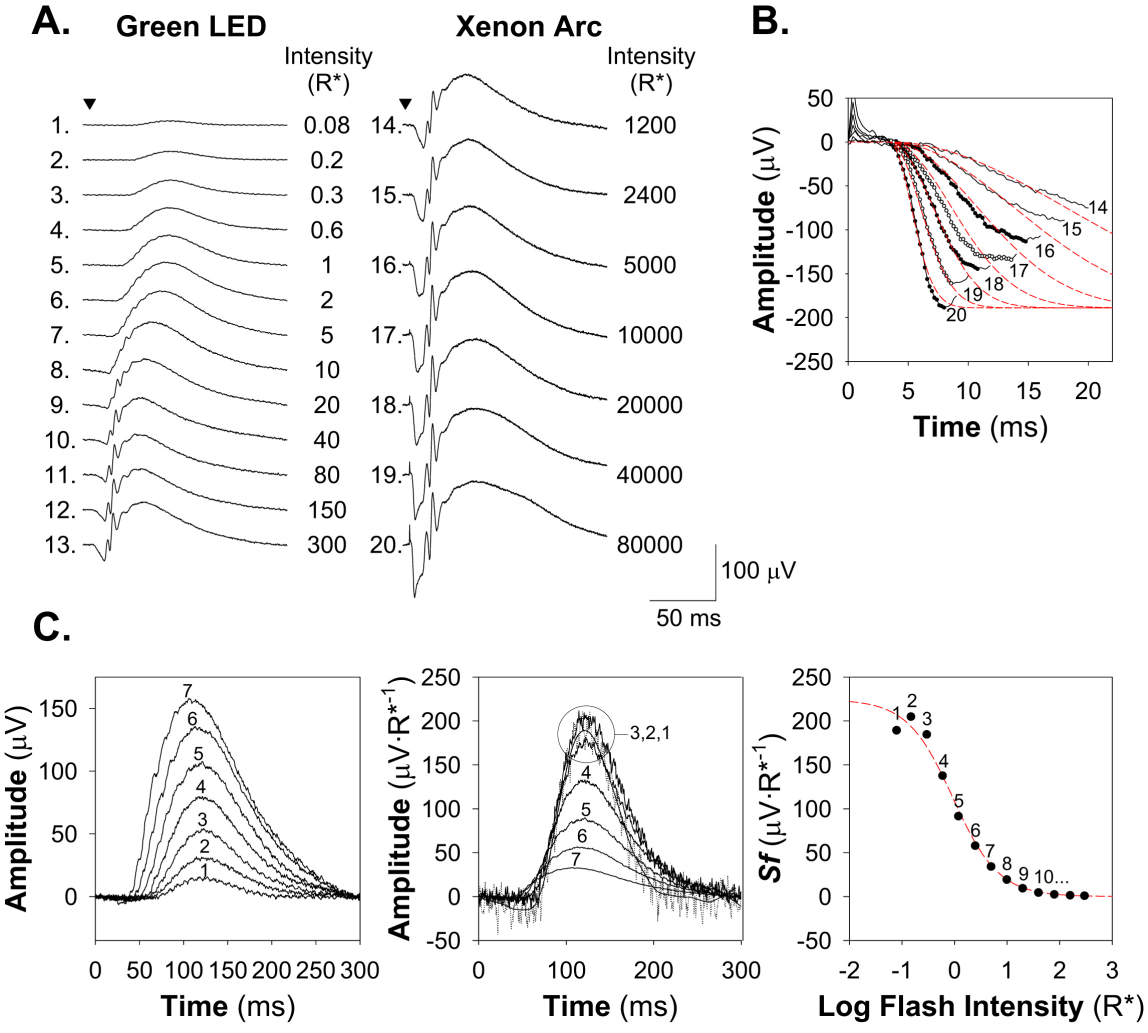
Electroretinographic (ERG) analyses of rod photoreceptor and postreceptor neural function. **A:** Sample ERG responses from a P25–P26 control rat elicited with green LED (left) and “white” xenon arc (right) full field stimuli. The number of rhodopsin photoisomerizations per rod (R*) produced by each flash is indicated at the end of each trace. Traces are numbered for reference in panels **B** and **C**. **B:** Determination of rod photoreceptor function. Sensitivity of the rods, *S*_rod_, is determined by fit of *Equation 3* (red dashes) to the leading edge of ERG a-waves (circles) elicited with bright flashes. For this subject, *S*_rod_ was 3.79 R*^−1^∙s^−2^. **C:** Determination of postreceptor function. The b-waves elicited by dim flashes (left panel) were scaled by the intensity of the flash used to elicit them (middle panel) and the Michaelis–Menten equation with the exponent set to −1 (*Equation 4*) fit to the resulting scaled response amplitudes (right panel). The limit of the function as intensity approaches zero, *Sm*, defines postreceptor sensitivity. For this subject, *Sm* was 224 µV∙R*^−1^.

Sample ERG responses are shown in Figure 1A. The a-wave results from the suppression of the circulating current of the photoreceptors.

Rod function was evaluated by ensemble fitting the Hood and Birch formulation [49] of the Lamb and Pugh model [50,51] of the activation of phototransduction to the leading edge of ERG a-waves elicited by five bright white flashes (Figure 1B). The model takes flash intensity, *i* (R*) and elapsed time, *t* (sec), as its inputs, such that

P3(i,t)=(1−exp⁡(−12×Srod×i×(t−td)2))×RmP3, for *t*_d_ < *t* < 20 ms, *Equation* 3

where *S*_rod_ is the sensitivity measure (R*^−1^∙sec^−2^), *t*_d_ is a delay of approximately 3.5 ms, and *Rm*_P3_ is the saturated amplitude of the photoreceptor response (μV). Fitting was restricted to the a-wave trough. *S*_rod_ summarizes the amplification time constants involved in the activation of phototransduction.

#### Analysis of postreceptor function

The amplitude of the b-wave was measured from the trough of the a-wave to the peak. At low intensities, under dark adapted conditions, the b-wave reflects mainly the activity of the rod bipolar cells (Figure 1C) [52,53]. Sensitivity of the b-wave is defined in the linear range of the response/intensity relationship as the amplitude of the b-wave scaled by stimulus intensity [54]. The b-wave amplitude increases in linear proportion to stimulus intensity over a narrow range of dim flash intensities [55]. Therefore, we calculated the sensitivity at threshold by scaling the amplitude of each b-wave by the intensity used to elicit it and fitting:

Sf(i)Sm=i−1i−1+i12*Equation* 4

to the resulting sensitivities. *Sf* (µV∙R*^−1^) is the fractional sensitivity of the b-wave response to a flash of *i* intensity, *Sm* (µV∙R*^−1^) is the sensitivity of the postreceptor retina at threshold, and *i_1/2_* (R*) is the stimulus intensity at which sensitivity has fallen to half that at threshold.

### Analysis of retinal vessels

In the same experimental sessions, digital photographs of the fundi of both eyes were obtained (RetCam; Clarity Medical Systems Inc., Dublin, CA). Several images were assembled into a composite (Photoshop CS3; Adobe Systems Inc., San Jose, CA) to create a complete view of the posterior pole, defined as the region within the circle bounded by the vortex veins and concentric to the optic nerve head (ONH). The arterioles were identified and their tortuosity measured using Retinal Image multi-Scale Analysis (RISA) software as previously described in detail [14]. In summary, arterioles were cropped from the main image and segmented individually. The segmented image was manually edited to remove extraneous features such as the background choroidal vasculature. RISA constructed a vessel skeleton from which the integrated curvature of the arteriole was measured. Integrated curvature, the sum of angles along a vessel normalized by its length, has demonstrated good agreement with human observer assessments of arteriolar tortuosity [56]. Arteriolar tortuosity, *T*_A_ (radians∙pixel^−1^), was calculated for each subject as the mean integrated curvature of all measurable arterioles in both eyes (median 9).

### Analysis of growth factor expression

#### Tissue preparation

Rats were euthanized with approximately 100 mg∙kg^−1^ pentobarbital administered intraperitoneally. Their corneas were incised and both retinas extracted. These were pooled, flash frozen in liquid nitrogen, and stored at −80° C.

#### RT–PCR

RNA was extracted using an RNeasy Mini Kit (Qiagen, Valencia, CA). The quantity of RNA extracted was determined spectrophotometrically (SmartSpec 3000; Bio-Rad Laboratories Inc., Hercules, CA). cDNA was reverse transcribed from each RNA sample in triplicate to mitigate the effects of noise and error. In each of three sample tubes, a quantity of RNA solution containing 300 ng of RNA was added to a solution of 8 µl 5X iScript Reaction Mix and 2 µl iScript Reverse Transcriptase (iScript cDNA Synthesis Kit; Bio-Rad). Nuclease-free water was then added to obtain a final volume of 40 µl per sample. Reverse transcription was achieved by incubating the mixtures at 25 °C for 5 min, 42 °C for 30 min, and 85 °C for 5 min. cDNA was stored at −20 °C.

PCR was performed on all three cDNA products employing primers (Table 1) for *VEGF_164_*, *NRP-1*, and *Sema3A* and using an appropriate temperature gradient. In addition, glyceraldehyde 3-phosphate dehydrogenase (*GAPDH*) served as the internal control.

**Table 1:** PCR primers

**Target**	**Primer sequence (5′-3′)**	**Annealing temp (°C)**	**Product length (bp)**
*VEGF_164_*	F: AACCATGAACTTTCTGCTCTC	52.8	635
	R: TTGTCACATCTGCAAGTACG	52.8	
*NRP-1*	F: GAAGGAGGGAAATAAAGCCA	52.3	572
	R: CTGATGAATCTTGTGGAGAG	50.2	
*Sema3A*	F: GACAACTTTCCTGAAAGCAC	51.9	535
	R: CCACTTTAAGGACAGTTCCA	52.1	
*GAPDH*	F: CATGTTCCAGTATGACTCTACC	52.3	829
	R: GTTGCTGTAGCCATATTCATTGTC	54.3	

The table provides key details of the primers used in the RT–PCR experiments. The growth factors studied were vascular endothelial growth factor 164 (*VEGF_164_*), semaphorin IIIA (*Sema3A*), and neuropilin-1 (*NRP-1*); glyceraldehyde 3-phosphate dehydrogenase (*GAPDH*) served as the internal control. The length of the product of the reaction is given in base pairs (bp).

Each reaction contained 3.0 µl cDNA and the following reagents (Bio-Rad): 5.0 µl 10x iTaq buffer, 1.5 µl 50 mM MgCl_2_, 1.0 µl 10 mM dNTP mix, 0.25 µl iTaq DNA polymerase, 15 µl 10 mM forward primer, 15 µl 10 mM reverse primer, and 9.25 µl sterile water for a total volume of 50 µl per reaction. The linear range of each target was determined empirically by increasing the number of cycles and resolving the products on a 2% agarose gel (Bio-Rad). The 12 products of RT–PCR (three each for *VEGF_164_*, *NRP-1*, *Sema3A*, and *GADPH*) and a molecular ruler (EZ-Load 100 bp; Bio-Rad) were resolved on a single 2% agarose gel. Gel imaging was performed using a GEL Logic 100 imaging system with Stratagene Transilluminator 2040 EV (Kodak Scientific Imaging Systems, New Haven, CT). The optical density of each band was determined (ImageJ version 1.38x; NIH, Bethesda, MD). The optical density of each growth factor band was divided by the optical density of its corresponding control gene (*GAPDH*) band. The two ratios in closest agreement for each growth factor were averaged in subsequent analyses.

#### Selection of control gene

Though commonly employed as an internal standard in experimental OIR, *GAPDH* expression is reportedly altered in severe hypoxia [57]. To assure the suitability of *GAPDH* as a control gene in the OIR models, we ran a pilot study on three 50/10 model, three 75 model, and three control rats (aged P15–16). *β-Actin* mRNA and *18S* rRNA expression, as well as *GAPDH*, were measured in this study. The ratio of the former genes expression to *GAPDH* was taken. The expression ratios were nearly constant across group (<0.1 log unit maximum difference). Specifically, in 75 model rats the expression of *β-Actin* and *18S* were each approximately +0.01 log unit relative to controls; between 50/10 model and control rats, the change was approximately +0.1 log unit for *β-Actin* and −0.1 log unit for *18S*. Thus, *GAPDH* appears to be an appropriate control gene in our experiment.

### Data analyses

All data were expressed as ΔLogNormal for the P25–26 controls:

$$\text{ΔLogNormal}\left(x\right)=\text{log}\left(x\right)-\frac{\sum_{rat=1}^{n}\log\left(\text{P25-26}\;\text{CTL}\;rat\right)}{n}$$

By expressing the data in log values, changes in observations of fixed proportion, either up or down, become linear. This is consistent with a constant fraction for physiologically meaningful changes in parameter values. Normalization by the mean of controls at P25–P26 allowed evaluation of changes with age. All parameters (*S*_rod_, *Sm*, *T*_A_, and mRNA expression of *VEGF_164_*, *NRP-1*, and *Sema3A*) were evaluated by ANOVA with factors age (P15–P16, P18–P19, P25–P26) and group (50/10 model, 75 model, control). Posthoc testing was performed using Tukey’s honestly significant difference (*q*) statistical test. Relations between parameters were evaluated by Pearson product moment correlation. The significance level (α) for all tests was p<0.01.

## Results

**Figure 2:**
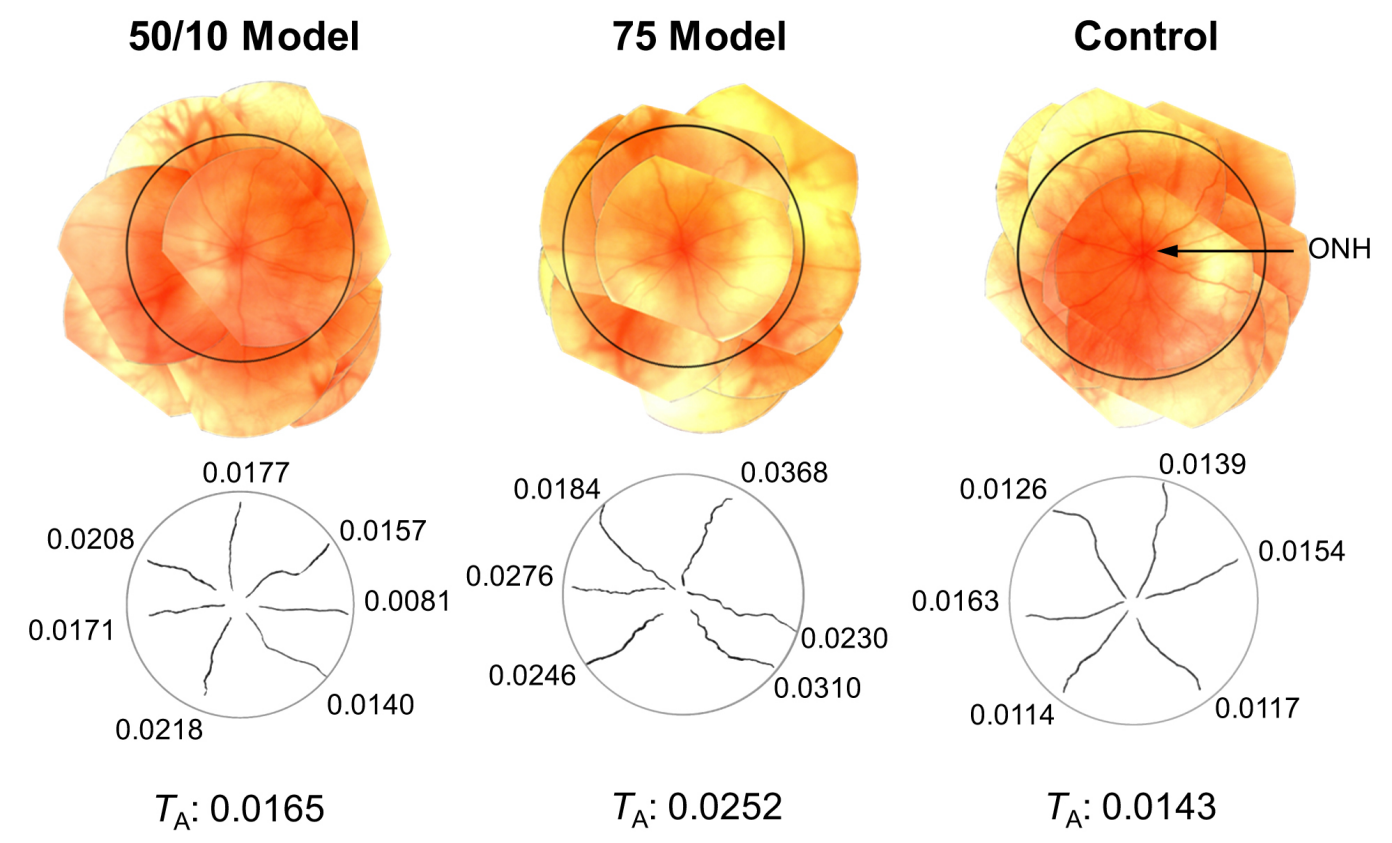
Composite RetCam images (top panels) and the retinal arterioles as segmented by RISA (bottom panels). The images were obtained from a 50/10 model, a 75 model, and a control rat at P25–26. The posterior pole, defined as the region bounded by the vortex veins and concentric to the optic nerve head, is indicated (circles). The integrated curvatures (radians∙pixel^−1^) corresponding to each segmented vessel are given. The mean tortuosity of the arterioles, *T*_A_ (radians∙pixel^−1^), for each eye is given at the bottom of the figure.

Figure 2 shows representative fundus images and the retinal arterioles as segmented by RISA from a 50/10 model, a 75 model, and a control rat imaged at P25–26. The average tortuosity of the arterioles, *T*_A_ (radians∙pixel), was highest in the 75 model rat and lowest in the control.

**Figure 3:**
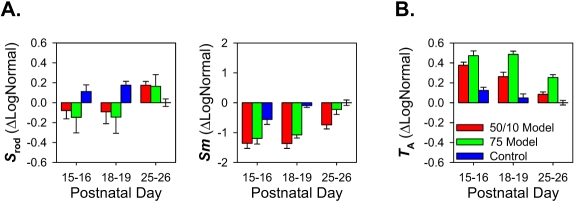
Mean±SEM ΔLogNormal (*Equation 5*) ERG and blood vessel parameters for 50/10 model, 75 model, and control rats at P15–P16, P18–P19, and P25–P26. Note that mean ΔLogNormal for P25–P26 control rats is zero. **A:** Sensitivity of the rod photoreceptors, *S*_rod_, and postreceptor neural retina, *Sm*. **B:** Arteriolar tortuosity, *T*_A_.

**Figure 4:**
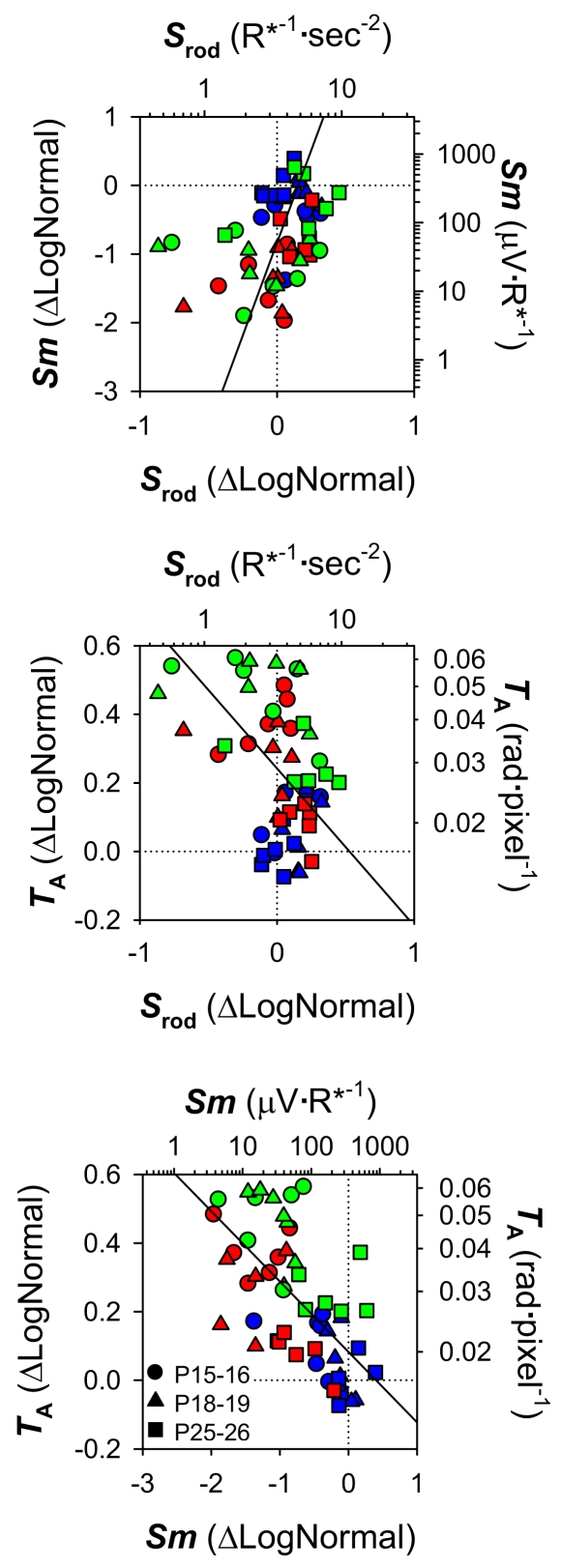
Relations of neural and vascular parameters for 50/10 model (red), 75 model (green), and control (blue) rats. Symbols indicate age at test (key). Postreceptor sensitivity, *Sm*, is plotted as a function of rod photoreceptor sensitivity, *S*_rod_ (top panel). Arteriolar tortuosity (*T*_A_) is plotted as a function of *S*_rod_ (middle panel) and *Sm* (bottom panel). The left ordinates and bottom abscissae are expressed in derived (*Equation 5*) ΔLogNormal units; the right ordinates and top abscissae are linear values. Stippled lines indicate the means for the P25–P26 controls. Solid lines are orthogonal linear regressions through all the data. Low *S*_rod_ was significantly related to low *Sm* (*r*=0.35; p=0.009) and high *T*_A_ (*r*=-0.40; p=0.003). Low *Sm* was also significantly related to high *T*_A_ (*r*=-0.62; p<0.001).

Figure 3 plots mean±SEM ΔLogNormal rod photoreceptor sensitivity, *S*_rod_, postreceptor sensitivity, *Sm*, and arteriolar tortuosity, *T*_A_. *S*_rod_ was not significantly affected by OIR or age. However, *Sm* was significantly affected by OIR (*F*=32.2; *df*=2,45; p<0.001), being more than 0.6 log unit below control values in both OIR models at early ages (P15–P16, P18–P19), but recovered significantly by P25–P26 (*F*=19.5; *df*=2,45; p<0.001). The results for *T*_A_ mirrored those for *Sm*, with *T*_A_ high when *Sm* was low and *T*_A_ becoming lower when *Sm* increased. *T*_A_ was high in OIR rats (*F*=73.4; *df*=2,45; p<0.001) at early ages but became significantly more normal by P25–P26 (*F*=29.0; *df*=2,45; p<0.001). Of note, *Sm* remained markedly low even at P25–P26 in 50/10 model rats, while *T*_A_ remained markedly high in 75 model rats. Figure 4 plots the three interrelations between these parameters (*S*_rod_ versus *Sm*, *S*_rod_ versus *T*_A_, and *Sm* versus *T*_A_) across age and group. All three parameters were correlated. Postreceptor sensitivity depends in part upon photoreceptor sensitivity, and these parameters (*S*_rod_, *Sm*) were positively correlated. Consistent with previous findings in OIR rats, high *T*_A_ correlated with low *S*_rod_. However, the value of the correlation coefficient was larger between *Sm* and *T*_A_ than between *S*_rod_ and *T*_A_. Indeed, 33 of the 36 OIR rats tested had reduced postreceptor sensitivity and increased vascular abnormalities relative to the mean for P25–P26 controls, and 29 fell outside the range of values observed in the controls at any age.

**Figure 5:**
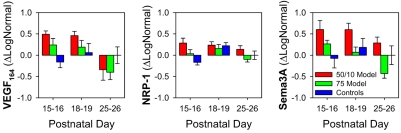
Mean±SEM ΔLogNormal (Equation 5) mRNA expression. Growth factor expression data for the 50/10 model, 75 model, and control rats at P15–16, P18–19, and P25–26 are shown. Note that mean ΔLogNormal for P25–26 control rats is zero.

Figure 5 plots mean±SEM ΔLogNormal growth factor mRNA expression for *VEGF_164_*, *NRP-1*, and *Sema3A*. Expression of the ligands *VEGF_164_* and *Sema3A* was significantly altered in OIR rats. In both OIR models, *VEGF_164_*, mainly associated with angiogenesis, was elevated at early ages (P15–P16, P18–P19) and decreased significantly with age (*F*=7.5; *df*=2,45; p=0.002) to levels well below normal at P25–P26. In control rats, *VEGF_164_* changed little with age. *Sema3A*, mainly associated with neurogenesis, was significantly elevated in 50/10 model rats (*F*=8.4; *df*=2,45; p=0.001); 75 model rats did not significantly differ from controls. *NRP-1*, a receptor for both *VEGF_164_* and *Sema3A*, displayed no significant effect of group or age (nor a group by age interaction).

**Figure 6:**
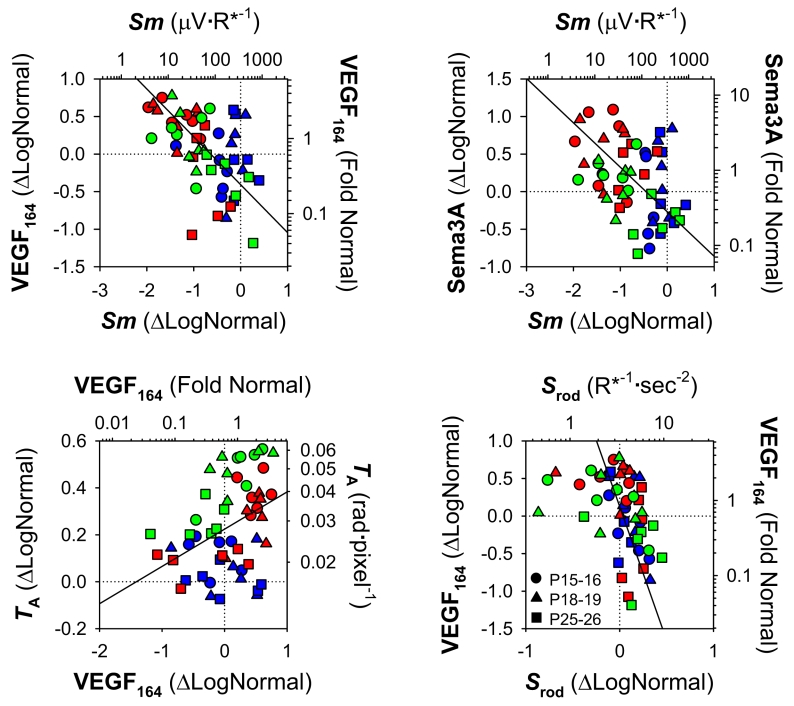
Significant relations between growth factor mRNA expression, photoreceptor and post-receptor sensitivity, and arteriolar tortuosity. Results from RT–PCR (*VEGF_164_, Sema3A*), ERG (*S_rod_, Sm*), and RISA (*T*_A_) analyses for 50/10 model (red), 75 model (green), and control (blue) rats are plotted with different symbols indicating age at test (key). The left ordinates and bottom abscissae are expressed in derived (Equation 5) ΔLogNormal units; the right ordinates and top abscissae are linear values. Stippled lines indicate the means for the P25-P26 controls. Solid lines are orthogonal linear regressions through all the data. Low *Sm* was significantly associated with high *VEGF_164_* (*r*=-0.49; p<0.001) and *Sema3A* expression (*r*=-0.38; p=0.005). Elevated *VEGF_164_* was related to high *T*_A_ (*r*=0.35; p=0.009). Low *S_rod_* was significantly related to high *VEGF_164_* (*r*=-0.39; p=0.003).

As shown in Figure 6, across group and age, deficits in postreceptor sensitivity (*Sm*) were negatively correlated with *VEGF_164_* and *Sema3A* mRNA expression. *NRP-1* receptor mRNA was weakly negatively associated with postreceptor sensitivity (p=0.011, not shown). Overexpression of *VEGF_164_* is documented in OIR models and is also known to promote pathological angiogenesis [58,59]. High expression of *VEGF_164_* mRNA was a significant predictor of high *T*_A_. *S*_rod_ was, in turn, negatively correlated with *VEGF_164_* expression; that is, poor rod function was associated with high *VEGF_164_* mRNA.

## Discussion

In these rats with OIR, the function of the postreceptor neural retina was significantly altered. Postreceptor sensitivity, *Sm*, was low in both OIR models at P15–P16 but recovered with age (Figure 3). These age-related improvements in neural function were accompanied by improvements in the vascular parameter, *T*_A_. In addition, we found that *Sm*, which measures postreceptor sensitivity at threshold, was correlated with *T*_A_ in these OIR rats (Figure 4). Rod photoreceptor sensitivity, *S*_rod_, was also correlated with *T*_A_, though the strength of the correlation was weaker. The mRNA expression of growth factors mediating both angiogenesis and neurogenesis, expressed relative to normal, was also altered (Figure 5). We found that mRNA of *VEGF_164_* was elevated in OIR rats, in agreement with other reports [58,59]. To our knowledge, this is the first report of alterations in a “neural” growth factor in OIR; *Sema3A* mRNA expression was upregulated in 50/10 model rats.

In ROP, the severity of consequent vascular abnormality depends upon the extent of earlier rod photoreceptor dysfunction, although the rods and the retinal blood vessels are anatomically separated. We replicated the finding [14] that low rod sensitivity (*S*_rod_) predicts abnormal blood vessels (*T*_A_; Figure 4). *S*_rod_ was, in turn, negatively correlated with *VEGF_164_* expression (Figure 6). The postreceptor neural retina is driven by rod photoreceptors in the outer retina, and is supplied by the retinal vasculature that traverses the inner retinal surface, sending capillaries deep into the inner neural layers. Thus, the postreceptor neural retina may be the bridge between rods and retinal vasculature and is in a position to mediate the rod and retinal vascular relation (Figure 4). Consistent with this position, in these data, postreceptor sensitivity was significantly associated not just with rod function and vascular abnormality (Figure 4), but also with mRNA expression of *VEGF_164_* and *Sema3A* (Figure 6).

There are at least two explanations for the strong negative relation between *Sm* and *T*_A_: 1) the pathologic vasculature associated with high values for *T*_A_ presumably adversely affects the retinal circulation, while low values for *T*_A_ putatively represent a salubrious postreceptor environment favorable to *Sm*; 2) the distressed postreceptor neural retina may signal the need for improved circulation by upregulating mRNA of proangiogenic growth factors such as *VEGF* or semaphorins. An excess of these signals may induce the pathologic vasculature that resulted in high *T*_A_. These explanations are not necessarily mutually exclusive. However, although *Sema3A* expression was markedly altered by OIR, it was not a significant predictor of blood vessel tortuosity.

Since rod photoreceptor dysfunction antedates and predicts consequent vascular abnormality [14], rod dysfunction may underpin both postreceptor neural dysfunction and retinal vascular abnormality. Deficits in postreceptor sensitivity in OIR are presumably due, in part, to diminished rod signaling, but may be worsened by direct insult to the rod bipolar cell, such as from hypoxia consequent to an avascularized inner retina and exacerbated by oxygen-demanding neighboring rods. Natural responses in the postreceptor retina would be to: 1) remodel neuron-to-neuron connections to enhance sensitivity; and 2) promote vascular development. Both outcomes could be achieved through the mediation of growth factors such as *VEGF*s and semaphorins expressed by intermingled glia [60,61].

Two retinal targets for intervention are suggested by these data, the molecular crosstalk between postreceptor neurons and their vasculature and the immature rod photoreceptors themselves. Mitigation of *VEGF*, either by the reduction of its expression or by antagonism of its receptors, has garnered much attention [62–66]. Successful treatment of the retinal vasculature would result in enhanced vascular supply to the postreceptor neurons and presumably improve visual function. However, in our data, at early ages when postreceptor sensitivity was low, *VEGF* expression was elevated. As postreceptor sensitivity recovered, *VEGF* expression plummeted. Endogenous *VEGF* is required for visual function [67]. Possibly, the high *VEGF* expression instigated successful receptor remodeling. Indeed, anti-*VEFG* pharmaceuticals have the potential for adverse effects on developing neurons [67–69], and thus any anti-*VEGF* therapy in ROP calls for caution. Moreover, since neural dysfunction antedates the vascular abnormalities [13], it seems unlikely that anti-*VEGF* therapy alone can fully normalize retinal function in ROP.

If the rods mediate the neurosensory dysfunction in ROP, then treatments that protect the rods may also protect the postreceptor neurons and would possibly reduce *VEGF* expression. Thus, treatments designed to relieve the burgeoning aerobic demands of the immature rods during the ages when the rod outer segments are elongating should be among the treatments considered for ROP. Simple approaches, such as treatment with light to suppress the circulating current [70], have shown small beneficial effect in OIR rats [71]. Pharmaceutical protection of the immature rods therefore represents a promising, though untested, approach to the management of ROP [72].

It should be noted that several recent approaches may derive some of their efficacy from neuroprotective effects. For example, omega-3 polyunsaturated fatty acids (ω3-PUFAs) increased vessel regrowth after the induction of retinopathy in a mouse model of ROP, reducing consequent pathologic vascularization [73]. One action of ω3-PUFAs is to lower inflammatory cytokine production in retinal microglia in the inner retina, directly mediating angiogenesis. However, ω3-PUFAs are also neuroprotective during ischemia [74], suggesting that some of their effect on the retinal vasculature may, in fact, be due to preservation of rod function. Unfortunately, we are unaware of any assessments of retinal function that have been applied, to date, in an antiangiogenic treatment in OIR models, leaving the role of the rods and postreceptor neurons equivocal.
